# A Novel Synthesis of Highly Functionalized Pyridines by a One-Pot, Three-Component Tandem Reaction of Aldehydes, Malononitrile and *N*-Alkyl-2-cyanoacetamides under Microwave Irradiation

**DOI:** 10.3390/molecules23030619

**Published:** 2018-03-09

**Authors:** Ramadan Ahmed Mekheimer, Mariam Abdullah Al-Sheikh, Hanadi Yousef Medrasi, Najla Hosain Hassan Alsofyani

**Affiliations:** 1 Chemistry Department, Faculty of Science, Minia University, Minia 61519, Egypt; 2 Chemistry Department, Faculty of Sciences-Al Faisaliah, King Abdulaziz University, Jeddah 21493, Saudi Arabia; r1425@hotmail.com (M.A.A.-S.); h.midrassi@gmail.com (H.Y.M.); chem.online@hotmail.com (N.H.H.A.)

**Keywords:** synthesis, one-pot, three-component, tandem reaction, microwave irradiation, pyridines

## Abstract

A convenient, fast and environmentally benign procedure for the synthesis of a new series of highly functionalized *N*-alkylated pyridines as privileged medicinal scaffolds was developed via a unique three-component reaction of easily available aromatic as well as heteroaromatic aldehydes, *N*-alkyl-2-cyanoacetamides and malononitrile in EtOH in the presence of K_2_CO_3_ as a base promoter under microwave irradiation. The presented tandem process is presumed to proceed via Knoevenagel condensation, Michael addition, intramolecular cyclization, autoxidation and subsequent aromatization. Particularly valuable features of this protocol, including high product yields, mild conditions, atom-efficiency, simple execution, short reaction times and easy purification make it a highly efficient and promising synthetic strategy to prepare substituted pyridine nuclei. The proposed mechanism of this novel one-pot reaction and structure elucidation of the products are discussed.

## 1. Introduction

One-pot multi-component reactions (MCRs) in which three or more reactants are combined together in a single synthetic operation to create a highly complex molecule incorporating most atoms present in the starting materials have proven to be a very rapid, powerful and elegant synthetic procedure. The MCRs strategy provides important advantages over conventional multistep synthesis because of its ease of execution, efficiency, simple procedures and equipment, flexibility, atom economic nature, high yields, productivity, convergence, and highly selectivity [1,2,3,4]. In addition, by reducing waste production, the number of operational steps, avoiding the complicated isolation and purification of intermediates, minimization of time, energy consumption, cost, solvents, reagents and expenditure of human labor, MCRs represent eco-friendly processes [5]. These advantages make MCRs well-suited for the easy construction of libraries of ‘drug-like’ molecules [6,7]. In view of the growing interest in the preparation of interesting heterocyclic scaffolds, tremendous scientific efforts are currently being devoted to develop new multi-component procedures for the synthesis of numerous polyfunctionalized heterocyclic scaffolds and discovery of new drugs [6].

Microwave-assisted organic chemistry (MAOC) is one of the high-speed techniques which has attracted a great attention in recent years. The intrinsic advantages of performing various organic transformations under microwave (MW) irradiation conditions are the high yields of relatively pure products and significant acceleration of the rate of the chemical reactions [8,9]. Thus, these are not only environmental friendly but also financially attractive processes [10].

Highly substituted pyridines, known as privileged medicinal scaffolds, are of significant interest as they widely occur as the key constituents in numerous of biologically active natural products and pharmaceuticals [11,12,13,14,15,16,17]. On account of their vast range of eminent pharmacological, physiological, and biological activities, they are considered important structures. Therefore, they have attracted great interest among the all heterocyclic compounds and the interest in their synthesis and chemistry continues undiminished [2,18,19]. Among these pyridine derivatives, 2-aminopyridine-3,5-dicarbonitriles constitute a very important type of heterocyclic compounds in modern medicinal chemistry due to their potential therapeutic applications in the treatment of several diseases and broad spectrum biological activities [20,21,22,23,24,25,26,27,28]. On the other hand, the *N*-alkylated pyridones are among the most important classes of azaheterocyclic compounds as they widely occur as prevalent core structures in many biologically active natural products, synthetic bioactive substances and active pharmaceuticals [29] that show interesting pharmacological and biological activities such as multiple sclerosis immunomodulators [30], a putative memory-enhancing drug [31,32], and anticancer agents [33]. Accordingly, methods for the efficient synthesis of new derivatives of these compounds have thus attracted the great interest of synthetic and medicinal chemists. However, a literature survey showed that efficient, direct approaches to the selective synthesis of *N*-alkylated 2-pyridone derivatives are much less well explored, as known methods generally suffer from certain drawbacks such as the lack of generality or selectivity, poor yields, the use of expensive transition-metal catalysts and/or a competitive process between *N*- and *O*-alkylation (poor chemoselectivity) [34,35]. Therefore, the development of novel straightforward approaches to densely substituted *N*-alkylated 2-pyridones still remains as a hot research topic. 

In the continuation of our efforts towards performing new synthetic methods for a wide variety of heterocycles under green conditions [36,37,38,39,40,41,42,43,44,45]. We report a general and efficient microwave-assisted one-pot three-component synthesis of a series of dense substituted *N*-alkylated 2-pyridones, utilizing malononitrile, a wide range of aromatic as well as heteroaromatic aldehydes and variety of *N*-alkyl-2-cyanoacetamides as building blocks. To the best of our knowledge, there are no reports in the literature on the synthesis of these compounds. Herein, we also report our experimental results using both thermal heating and microwave irradiation methods and we have compared our results, which shows the advantage of the microwave irradiation method. The proposed reaction mechanism is also discussed.

## 2. Results and Discussion

**Scheme 1:**
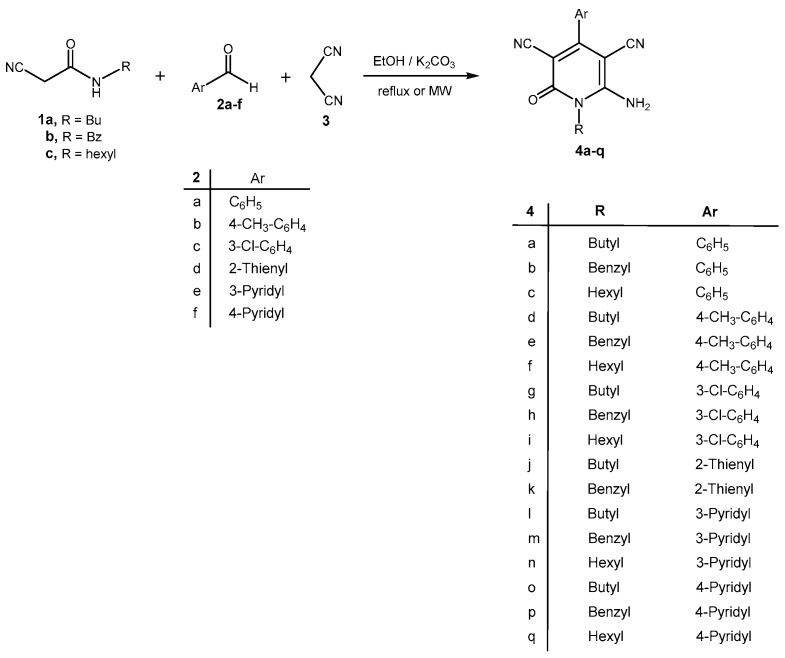
One-pot synthesis of 1-alkyl-6-amino-4-aryl(or het)-2-oxo-1,2-dihydropyridine-3,5-dicarbonitriles **4a**–**q**.

Initially, *N*-butyl-2-cyanoacetamide (**1a**), benzaldehyde (**2a**) and malononitrile (**3**) were adopted as simple model substrates for studying the multi-component synthesis of 1-alkyl-6-amino-4-aryl(or het)-2-oxo-1,2-dihydropyridine-3,5-dicarbonitriles. Indeed, after experimentation with different solvents, reaction temperatures and base catalysts, we found that the best result was obtained by stirring the solution of *N*-butyl-2-cyanoacetamide (**1a**, 4 mmol), benzaldehyde (**2a**, 4 mmol), and malononitrile (**3**, 4 mmol) in ethanol (7 mL) in the presence of K_2_CO_3_ (4 mmol) under reflux for one hour, whereupon after cooling and neutralization with HCl, a pale yellow solid was crystallized out. The precipitate was filtered, recrystallized from methanol and identified as the 6-amino-1-butyl-2-oxo-4-phenyl-1,2-dihydropyridine-3,5-dicarbonitrile (**4a**) (70% yield) (Scheme 1) (Table 1). The structure of the product **4a** was elucidated with the help of IR, ^1^H-NMR, ^13^C-NMR, mass spectral data, and elemental analyses. Its mass spectrum disclosed a molecular ion peak at *m*/*z* = 292 (M^+^) corresponding to the molecular formula C_17_H_16_N_4_O. The ^1^H-NMR spectrum of **4a** contained a triplet for CH_3_ (*δ* = 0.92), a multiplet for CH_2_ (*δ* = 1.34), a multiplet for CH_2_ (*δ* = 1.51), a triplet for N-CH_2_ (*δ* = 4.0), a multiplet for 2 × CHAr (*δ* = 7.48–7.49), a multiplet for 3 CHAr (*δ* = 7.54–7.55), and a singlet for NH_2_ (*δ* = 8.40). The assignment is supported by the IR absorptions at 3435, 3322, 3286, 3176 cm^−1^ (NH_2_), 2965, 2929 cm^−1^ (aliph. CH), 2212 cm^−1^ (CN), 1647 cm^−1^ (amide C=O). The proton-decoupled ^13^C-NMR spectrum of **4a** displayed 15 discreet resonances. Characteristic ^13^C-NMR signals due to C-5 and C-3 appeared at *δ* = 75.43 and 87.48 ppm, respectively, those of cyano carbons at *δ* = 115.90, 116.56 ppm and those of the C-6, C-2 and C-4 atoms at *δ* = 156.22, 159.35 and 160.36 ppm, respectively. All other aldehydes **2b**–**f** reacted analogously with *N*-alkyl-2-cyanoacetamides **1a**–**c** and malononitrile (**3**) under the same reaction conditions, leading to the formation of products **4b**–**q** in 65–77% yields as shown in Table 1 (Scheme 1). 

**Scheme 2:**
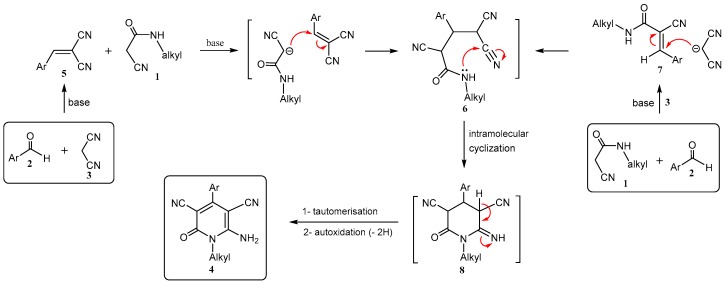
Plausible mechanisms for the synthesis of 1-alkyl-6-amino-4-aryl(or het)-2-oxo-1,2-dihydropyridine-3,5-dicarbonitriles **4a**–**q**.

For the formation of **4**, we propose two plausible mechanisms which are shown in Scheme 2. The process expresses a typical cascade reaction in which a Knoevenagel condensation between an aldehyde **2** and malononitrile (**3**) or *N*-alkyl-2-cyano-3-phenyl-acrylamide **1** and aldehyde **2** in the presence of K_2_CO_3_ as a base catalyst leads to the formation of 2-arylidenemalononitrile (Knoevenagel reagents) **5** and *N*-alkyl-3-aryl-2-cyano-acrylamide **7**, respectively. Then, Michael addition of the active methylene group of **1** to the activated double bond in **5** (or **3** to **7**) gives the non-isolable adduct **6**, which underwent an in situ cyclization via intramolecular addition of the amide nitrogen atom, as a nucleophile, to the nitrile function to give the intermediate **8**. The tautomerisation of the imino (=NH) function to the amino (-NH_2_) group followed by autoxidation and aromatization afforded the target product **4**. Thus, the reaction could proceed via a domino Knoevenagel condensation/Michael addition/intramolecular cyclization/autoxidation reaction sequence.

For the investigation of the reaction mechanism, both *Knoevenagel* reagents **5** and **7** were prepared from the reaction of aldehydes **2** with **3** or **1**, respectively, and then these were reacted with active methylene compounds **1** or **3**. The products **4** were again formed, but obtained in lower yields compared to our one-pot method, and longer reaction times were also required. 

In order to improve the yield and reduce the reaction times, we repeated the reaction of *N*-butyl-2-cyanoacetamide (**1a**), benzaldehyde (**2a**) and malononitrile (**3**) under microwave irradiation in EtOH in the presence of K_2_CO_3_ for 10 min at 90 °C (500 W, 200 rpm), whereupon **4a** was isolated in 91% yield. In order to demonstrate the scope of this reaction, a series of substituted aromatic as well as heteroaromatic aldehydes underwent this three-component condensation with different *N*-alkyl-2-cyanoacetamides and malononitrile by this procedure to give 1-alkyl-6-amino-4-aryl(or het)-2-oxo-1,2-dihydro-pyridine-3,5-dicarbo-nitriles. The results are summarized in Table 1. As is evident from the results shown in Table 1, this method is highly compatible with different aldehydes. Moreover, very good to high yields were also obtained for a heteroaromatic aldehydes when they were employed in this reaction. The microwave method was used in an effort to shorten reaction times and generate high yields. In addition, the analysis of the data in Table 1 indicates that the substituent on the aromatic aldehyde showed slightly different effects on the yields. Reactions of electron rich aromatic aldehydes afforded slightly better yields than electron deficient ones.

## 3. Experimental

### 3.1. General Information

All purchased solvents and chemicals were of analytical grade. Melting points were determined on a B-540 melting point apparatus (Büchi, Flawil, Switzerland) and are uncorrected. IR spectra were recorded on a Magna 520 FT-IR spectrophotometer (Nicolet, CA, USA) using potassium bromide disks and signals are reported in cm^−1^. ^1^H-NMR and ^13^C-NMR spectra were recorded on a DPX (850 MHz for ^1^H-NMR and 213 MHz for ^13^C-NMR) spectrometer (Bruker, Germany) using DMSO-*d*_6_ as a solvent, and TMS as an internal standard; the chemical shifts are given in δ units (ppm). Abbreviations used for NMR signals: *s* = singlet, *d* = doublet, *t* = triplet, and *m* = multiplet. Mass spectra were recorded on a Shimadzu (Kanagawa, Japan) mass spectrometer at 70 eV. All microwave irradiation experiments were carried out using a Monowave 300 Microwave Synthesis Reactor (MAS) equipped with a MAS 24 autosampler unit (Anton Paar GmbH, Graz, Austria). All experiments were carried out in 10 mL septum-capped microwave vials at 90 °C (500 W maximum power, 200 rpm). Microanalytical data were obtained from the Microanalytical Data Unit at Cairo University (Cairo, Egypt). 

### 3.2. General Procedure for the Synthesis of 1-Alkyl-6-amino-4-aryl(or het)-2-oxo-1,2-dihydro-pyridine-3,5-dicarbonitriles ***4a**–**q***

**Method I (∆)**. A mixture of *N*-alkyl-2-cyanoacetamides **1a**–**c** (4 mmol), aldehydes **2a**–**f** (4 mmol), malononitrile (**3**) (4 mmol), and K_2_CO_3_ (4 mmol) in refluxing EtOH (7 mL) was stirred for 1–4 h. Upon completion as monitored by TLC, the reaction mixture was cooled and poured into H_2_O. After neutralization with HCl, the resulting solid was filtered off, washed with H_2_O, dried and recrystallized from MeOH to give pure products **4a**–**q**.

**Method II (μω)**. A mixture of *N*-alkyl-2-cyano-acetamides **1a**–**c** (2 mmol), aldehydes **2a**–**f** (2 mmol), malononitrile (**3**) (2 mmol), K_2_CO_3_ (2 mmol), and EtOH (2 mL) in a 10 mL septum-capped microwave vials was irradiated under microwave conditions at 90 °C, 500 W, 200 rpm, for 10–15 min. After completion of the reaction, as indicated by TLC, each vial was de-capped and the contents were left to cool to room temperature. Then, the reaction mixture was worked up as described in method **I** to give compounds **4a**–**q**. Analytical samples were obtained by recrystallization from MeOH.

*6-Amino-1-butyl-2-oxo-4-phenyl-1,2-dihydropyridine-3,5-dicarbonitrile* (**4a**). Pale yellow crystals. M.p. 304–305 °C. IR (KBr) 3435, 3322, 3286, 3176 (NH_2_), 2965, 2929 (aliph. CH), 2212 (CN), 1647 (amide CO) cm^−1^. ^1^H-NMR (DMSO-*d*_6_) *δ* 0.92 (t, 3H, *J* = 6.8 Hz, CH_3_), 1.34 (m, 2H, CH_2_), 1.51 (m, 2H, CH_2_), 4.0 (t, 2H, *J* = 7.65 Hz, N-CH_2_), 7.48–7.49 (m, 2Ar-H), 7.54–7.55 (m, 3Ar-H), 8.40 (s, 2H, NH_2_). ^13^C-NMR (DMSO-*d*_6_) *δ* 13.74 (CH_2_), 19.31 (CH_2_), 28.38 (CH_2_), 41.87 (N-CH_2_), 75.43 (C-5), 87.48 (C-3), 115.90 (CN), 116.56 (CN), 127.98 (2Ar-C), 128.63 (2Ar-C), 130.25 (1Ar-C), 134.63 (1Ar-C), 156.22 (C-6), 159.35 (C-2), 160.36 (C-4). MS: *m*/*z* (%) = 293 (M^+^ + 1, 7), 292 (M^+^, 27), 276 (27), 275 (81), 250 (26), 237 (18), 236 (100), 235 (12), 209 (18), 208 (18), 180 (9), 165 (9), 77 (10); Anal. Calcd. for C_17_H_16_N_4_O (292.34): C, 69.85; H, 5.52; N, 19.17. Found: C, 69.73; H, 5.43; N, 18.99.

*6-Amino-1-benzyl-2-oxo-4-phenyl-1,2-dihydropyridine-3,5-dicarbonitrile* (**4b**). Pale yellow crystals. M.p. 251–252 °C. IR (KBr) 3439, 3318, 3182 (NH_2_), 3036 (arom. CH), 2961, 2877 (aliph. CH), 2225, 2215 (CN), 1656 (amide CO) cm^−1^. ^1^H-NMR (DMSO-*d*_6_) *δ* 5.35 (s, 2H, CH_2_), 7.25 (d, 2H, *J* = 7.65 Hz, Ar-H), 7.31 (t, 1H, *J* = 7.65 Hz, Ar-H), 7.38 (t, 2H, *J* = 7.65 Hz, Ar-H), 7.55–7.58 (m, 5Ar-H), 8.45 (s, 2H, NH_2_). ^13^C-NMR (DMSO-*d*_6_) *δ* 44.77 (N-CH_2_), 75.72 (C-5), 87.56 (C-3), 115.79 (CN), 116.48 (CN), 126.54 (2Ar-C), 127.45 (1Ar-C), 128.06 (2Ar-C), 128.58 (2Ar-C), 128.65 (2Ar-C), 130.34 (1Ar-C), 134.45 (1Ar-C), 134.61 (1Ar-C), 156.60 (C-6), 159.51 (C-2), 160.89 (C-4). MS: *m*/*z* (%) = 327 (M^+^ + 1, 6), 326 (M^+^, 25), 325 (8), 92 (8), 91 (100), 77 (3), 65 (15); Anal. Calcd. for C_20_H_14_N_4_O (326.36): C, 73.61; H, 4.32; N, 17.17. Found: C, 73.76; H, 4.40; N, 17.30.

*6-Amino-1-hexyl-2-oxo-4-phenyl-1,2-dihydropyridine-3,5-dicarbonitrile* (**4c**). Colorless crystals. M.p. 252–254 °C. IR (KBr) 3436, 3415, 3328, 3284, 3207 (NH_2_), 2933, 2869 (aliph. CH), 2209 (CN), 1653 (amide CO) cm^−1^. ^1^H-NMR (DMSO-*d*_6_) *δ* 0.88 (t, 3H, *J* = 6.8 Hz, CH_3_), 1.28–1.35 (m, 6H, 3CH_2_), 1.51–1.54 (m, 2H, CH_2_), 3.99 (t, 2H, *J* = 6.8 Hz, N-CH_2_), 7.48–7.49 (m, 2H, Ar-H), 7.54–7.55 (m, 3H, Ar-H), 8.41 (s, 2H, NH_2_). ^13^C-NMR (DMSO-*d*_6_) *δ* 13.94 (CH_3_), 22.03 (CH_2_), 25.59 (CH_2_), 26.20 (CH_2_), 31.0 (CH_2_), 42.13 (N-CH_2_), 75.40 (C-5), 87.47 (C-3), 115.89 (CN), 116.54 (CN), 127.99 (2Ar-C), 128.63 (2Ar-C), 130.24 (1Ar-C), 134.62 (1Ar-C), 156.20 (C-6), 159.33 (C-2), 160.35 (C-4). MS: *m*/*z* (%) = 321 (M^+^ + 1, 6), 320 (M^+^, 25), 305 (6), 304 (33), 303 (100), 263 (6), 261 (5), 250 (18), 237 (14), 236 (66), 235 (5), 220 (5), 209 (8), 208 (8), 165 (7), 77 (6), 69 (8), 57 (6), 56 (10), 55 (34); Anal. Calcd. for C_19_H_20_N_4_O (320.40): C, 71.23; H, 6.29; N, 17.49. Found: C, 71.16; H, 6.44; N, 17.38.

*6-Amino-1-butyl-2-oxo-4-(p-tolyl)-1,2-dihydropyridine-3,5-*dicarbonitrile (**4d**). Colorless crystals. M.p. 286–288 °C. IR (KBr) 3416, 3338, 3219 (NH_2_), 2953, 2932, 2873 (aliph. CH), 2205 (CN), 1653 (amide CO) cm^−1^. ^1^H-NMR (DMSO-*d*_6_) *δ* 0.91 (t, 3H, *J* = 6.8 Hz, CH_3_), 1.32–1.36 (m, 2H, CH_2_), 1.49–1.53 (m, 2H, CH_2_), 2.39 (s, 3H, CH_3_), 4.0 (t, 2H, *J* = 7.65 Hz, N-CH_2_), 7.34 (d, 2H, *J* = 7.65 Hz, Ar-H), 7.38 (d, 2H, *J* = 8.5 Hz, Ar-H), 8.38 (s, 2H, NH_2_). ^13^C-NMR (DMSO-*d*_6_) *δ* 13.72 (CH_3_), 20.98 (CH_2_), 21.50 (CH_3_), 28.38 (CH_2_), 41.84 (N-CH_2_), 75.35 (C-5), 87.37 (C-3), 115.99 (CN), 116.64 (CN), 127.97 (1Ar-C), 129.14 (1Ar-C), 130.20 (1Ar-C), 130.72 (1Ar-C), 131.68 (1Ar-C), 140.08 (1Ar-C), 156.20 (C-6), 159.36 (C-2), 160.37 (C-4). MS: *m*/*z* (%) = 307 (M^+^ + 1, 8), 306 (M^+^, 33), 290 (31), 289 (88), 264 (27), 251 (19), 250 (100), 249 (15), 236 (7), 235 (6), 234 (12), 233 (24), 223 (7), 222 (11), 221 (7), 207 (7), 206 (6), 205 (6), 194 (8), 180 (7), 179 (11), 140 (7), 91 (9), 77 (4), 65 (7), 57 (6), 56 (8), 55 (16); Anal. Calcd. for C_18_H_18_N_4_O (306.37): C, 70.57; H, 5.92; N, 18.29. Found: C, 70.47; H, 6.06; N, 18.22.

*6-Amino-1-benzyl-2-oxo-4-(p-tolyl)-1,2-dihydropyridine-3,5-dicarbonitrile* (**4e**). Colorless crystals. M.p. 303.9–305.9 °C. IR (KBr) 3322, 3143 (NH_2_), 2930, 2875 (aliph. CH), 2224, 2213 (CN), 1657 (amide CO) cm^−1^. ^1^H-NMR (DMSO-*d*_6_) *δ* 2.41 (s, 3H, CH_3_), 5.34 (s, 2H, N-CH_2_), 7.24 (d, 2H, *J* = 6.8 Hz, Ar-H), 7.31 (t, 1H, *J* = 6.8 Hz, Ar-H), 7.38 (t, 4H, *J* = 7.65 Hz, Ar-H), 7.44 (d, 2H, *J* = 8.5 Hz, Ar-H), 8.42 (s, 2H, NH_2_). ^13^C-NMR (DMSO-*d*_6_) *δ* 21.0 (CH_3_), 44.73 (CH_2_), 75.66 (C-5), 87.48 (C-3), 115.89 (CN), 116.58 (CN), 126.53 (2Ar-C), 127.43 (1Ar-C), 128.05 (2Ar-C), 128.58 (2Ar-C), 129.17 (2Ar-C), 131.67 (1Ar-C), 134.48 (1Ar-C), 140.22 (1Ar-C), 156.59 (C-6), 159.53 (C-2), 160.91 (C-4). MS: *m*/*z* (%) = 341 (M^+^ + 1, 7), 340 (M^+^, 28), 339 (7), 92 (8), 91 (100), 65 (14); Anal. Calcd. for C_21_H_16_N_4_O (340.39): C, 74.10; H, 4.74; N, 16.46. Found: C, 74.16; H, 4.65; N, 16.59.

*6-Amino-1-hexyl-2-oxo-4-(p-tolyl)-1,2-dihydropyridine-3,5-dicarbonitrile* (**4f**). Colorless crystals. M.p. 260.4–261.7 °C. IR (KBr) 3416, 3284, 3204 (NH_2_), 2965, 2927, 2857 (aliph. CH), 2210 (CN), 1652 (amide CO) cm^−1^. ^1^H-NMR (DMSO-*d*_6_) *δ* 0.88 (t, 3H, *J* = 6.8 Hz, CH_3_), 1.29–1.34 (m, 6H, 3CH_2_), 1.50–1.53 (m, 2H, CH_2_), 2.39 (s, 3H, CH_3_), 3.98 (t, 2H, *J* = 7.65 Hz, CH_2_), 7.34 (d, 2H, *J* = 8.5 Hz, Ar-H), 7.38 (d, 2H, *J* = 7.65 Hz, Ar-H), 8.37 (br s, 2H, NH_2_). ^13^C-NMR (DMSO-*d*_6_) *δ* 13.94 (CH_3_), 20.98 (CH_3_), 22.01 (CH_2_), 25.57 (CH_2_), 26.19 (CH_2_), 30.97 (CH_2_), 42.06 (N-CH_2_), 75.36 (C-5), 87.32 (C-3), 116.00 (CN), 116.66 (CN), 127.97 (2Ar-C), 129.14 (2Ar-C), 131.69 (1Ar-C), 140.08 (1Ar-C), 156.19 (C-6), 159.36 (C-2), 160.36 (C-4). MS: *m*/*z* (%) = 335 (M^+^ + 1, 8), 334 (M^+^, 25), 319 (5), 318 (27), 317 (78), 277 (6), 275 (5), 264 (26), 251 (22), 250 (100), 246 (10), 234 (9), 233 (15), 222 (7), 179 (6), 69 (7), 56 (9), 55 (33); Anal. Calcd. for C_20_H_22_N_4_O (334.42): C, 71.83; H, 6.63; N, 16.75. Found: C, 71.90; H, 6.57; N, 16.91.

*6-Amino-1-butyl-4-(3-chlorophenyl)-2-oxo-1,2-dihydropyridine-3,5-dicarbonitrile* (**4g**). Colorless crystals. M.p. 249.6–251.6 °C. IR (KBr) 3415, 3340, 3201 (NH_2_), 2959, 2872 (aliph. CH), 2213 (CN), 1655 (amide CO) cm^−1^. ^1^H-NMR (DMSO-*d*_6_) *δ* 0.92 (t, 3H, *J* = 6.8 Hz, CH_3_), 1.32–1.37 (m, 2H, CH_2_), 1.49–1.53 (m, 2H, CH_2_), 4.01 (t, 2H, *J* = 7.65 Hz, N-CH_2_), 7.46 (d, 1H, *J* = 6.8 Hz, Ar-H), 7.59 (t, 1H, *J* = 8.5 Hz, Ar-H), 7.60 (s, 1Ar-H), 7.63 (d, 1H, *J* = 8.5 Hz, Ar-H), 8.47 (s, 2H, NH_2_). ^13^C-NMR (DMSO-*d*_6_) *δ* 13.72 (CH_3_), 19.29 (CH_2_), 28.35 (CH_2_), 41.91 (N-CH_2_), 75.45 (C-5), 87.54 (C-3), 115.68 (CN), 116.32 (CN), 126.81 (1Ar-C), 127.78 (1Ar-C), 130.14 (1Ar-C), 130.71 (1Ar-C), 133.21 (1Ar-C), 136.61 (1Ar-C), 156.15 (C-6), 158.76 (C-2), 159.18 (C-4). MS: *m*/*z* (%) = 328 (M^+^ + 2, 11), 326 (M^+^, 33), 312 (11), 311 (37), 310 (34), 309 (100), 286 (8), 284 (23), 272 (29), 271 (18), 270 (87), 269 (8), 243 (16), 242 (10), 207 (13), 199 (6), 180 (15), 165 (7), 68 (5), 57 (10), 56 (15), 55 (24); Anal. Calcd. for C_17_H_15_ClN_4_O (326.78): C, 62.48; H, 4.63; Cl, 10.85; N, 17.15. Found: C, 62.40; H, 4.59; Cl, 10.96; N, 17.21.

*6-Amino-1-benzyl-4-(3-chlorophenyl)-2-oxo-1,2-dihydropyridine-3,5-dicarbonitrile* (**4h**). Pale yellow crystals. M.p. 231.8–233.3 °C. IR (KBr) 3643, 3471, 3331, 3193 (NH_2_), 3062 (arom. CH), 2987 (aliph. CH), 2225, 2212 (CN), 1661 (amide CO) cm^−1^. ^1^H-NMR (DMSO-*d*_6_) *δ* 5.34 (s, 2H, CH_2_), 7.23 (d, 2H, *J* = 7.65 Hz, Ar-H), 7.31 (t, 1H, *J* = 7.65 Hz, Ar-H), 7.38 (t, 2H, *J* = 7.65 Hz, Ar-H), 7.53 (d, 1H, *J* = 7.65 Hz, Ar-H), 7.61 (t, 1H, *J* = 7.65 Hz, Ar-H), 7.64–7.65 (m, 1Ar-H), 7.68 (s, 1Ar-H), 8.50 (br s, 2H, NH_2_). ^13^C-NMR (DMSO-*d*_6_) *δ* 44.75 (CH_2_), 75.77 (C-5), 87.65 (C-3), 115.59 (CN), 116.27 (CN), 126.53 (2Ar-C), 126.84 (1Ar-C), 127.46 (1Ar-C), 127.86 (1Ar-C), 128.56 (2Ar-C), 130.20 (1Ar-C), 130.72 (1Ar-C), 133.21 (1Ar-C), 134.33 (1Ar-C), 136.62 (1Ar-C), 156.52 (C-6), 159.29 (C-2), 159.33 (C-4). MS: *m*/*z* (%) = 362 (M^+^ + 2, 4), 360 (M^+^ + 12), 92 (8), 91 (100), 65 (13); Anal. Calcd. for C_20_H_13_ClN_4_O (360.80): C, 66.58; H, 3.63; Cl, 9.83; N, 15.53. Found: C, 66.67; H, 3.76; Cl, 9.67; N, 15.46.

*6-Amino-4-(3-chlorophenyl)-1-hexyl-2-oxo-1,2-dihydropyridine-3,5-dicarbonitrile* (**4i**). Colorless crystals. M.p. 235.9–236.6 °C. IR (KBr) 3423, 3292, 3180 (NH_2_), 3079 (arom. CH), 2954, 2934, 2869 (aliph. CH), 2214 (CN), 1645 (amide CO) cm^−1^. ^1^H-NMR (DMSO-*d*_6_) *δ* 0.88 (t, 3H, *J* = 6.8 Hz, CH_3_), 1.28–1.35 (m, 6H, 3CH_2_), 1.50–1.53 (m, 2H, CH_2_), 3.99 (t, 2H, *J* = 7.65 Hz, N-CH_2_), 7.46 (d, 1H, *J* = 7.6 Hz, Ar-H), 7.58 (d, 1H, *J* = 7.65 Hz, Ar-H), 7.60 (s, 1Ar-H), 7.62–7.63 (m, 1Ar-H), 8.46 (br s, 2H, NH_2_). ^13^C-NMR (DMSO-*d*_6_) *δ* 13.95 (CH_3_), 22.02 (CH_2_), 25.55 (CH_2_), 26.16 (CH_2_), 30.98 (CH_2_), 42.13 (N-CH_2_), 75.44 (C-5), 87.53 (C-3), 115.67 (CN), 116.32 (CN), 126.8 (1Ar-C), 127.76 (1Ar-C), 130.14 (1Ar-C), 130.71 (1Ar-C), 133.20 (1Ar-C), 136.61 (1Ar-C), 156.13 (C-6), 158.75 (C-2), 159.17 (C-4). MS: *m*/*z* (%) = 356 (M^+^ + 2, 9), 354 (M^+^, 26), 340 (11), 339 (35), 338 (32), 337 (94), 297 (8), 286 (8), 284 (24), 273 (7), 272 (34), 271 (23), 270 (100), 269 (7), 243 (11), 242 (7), 180 (8), 69 (12), 56 (19), 55 (51); Anal. Calcd. for C_19_H_19_ClN_4_O (354.84): C, 64.31; H, 5.40; Cl, 9.99; N, 15.79. Found: C, 64.42; H, 5.47; Cl, 10.14; N, 15.71.

*6-Amino-1-butyl-2-oxo-4-(thiophen-2-yl)-1,2-dihydropyridine-3,5-dicarbonitrile* (**4j**). Yellow crystals. M.p. 264.4–265.9 °C. IR (KBr) 3408, 3327, 3285, 3223 (NH_2_), 2959, 2940, 2874 (aliph. CH), 2207 (CN), 1634 (amide CO) cm^−1^. ^1^H-NMR (DMSO-*d*_6_) *δ* 0.91 (t, 3H, *J* = 6.8 Hz, CH_3_), 1.33 (m, 2H, CH_2_), 1.51 (m, 2H, CH_2_), 3.99 (t, 2H, *J* = 7.65, N-CH_2_), 7.26 (dd, 1H, *J* = 3.4, 3.4 Hz, thiophene-H), 7.51 (dd, *J* = 1.7, 0.85 Hz, thiophene-H), 7.91 (dd, *J* = 1.7, 1.7 Hz, thiophene-H), 8.41 (br s, 2H, NH_2_). ^13^C-NMR (DMSO-*d*_6_) *δ* 13.73 (CH_3_), 19.3 (CH_2_), 28.31 (CH_2_), 41.95 (N-CH_2_), 75.37 (C-5), 87.42 (C-3), 116.04 (CN), 116.64 (CN), 127.72 (thiophene-C), 130.32 (thiophene-C), 130.79 (thiophene-C), 133.37 (thiophene-C), 152.45 (C-6), 156.31 (C-2), 159.30 (C-4). MS: *m*/*z* (%) = 299 (M^+^ + 1, 8), 298 (M^+^, 34), 283 (7), 282 (27), 281 (81), 269 (7), 256 (24), 244 (6), 243 (18), 242 (100), 241 (7), 228 (7), 215 (13), 214 (19), 213 (6), 208 (12), 198 (5), 185 (7), 182 (9), 176 (9), 171 (8), 160 (7), 159 (6), 69 (11), 58 (8), 57 (11), 56 (10), 55 (21); Anal. Calcd. for C_15_H_14_N_4_OS (298.36): C, 60.38; H, 4.73; N, 18.78; S, 10.75. Found: C, 60.30; H, 4.68; N, 18.89; S, 10.89.

*6-Amino-1-benzyl-2-oxo-4-(thiophen-2-yl)-1,2-dihydropyridine-3,5-dicarbonitrile* (**4k**). Pale yellow crystals. M.p. 236.8–238.8 °C. IR (KBr) 3444, 3303, 3215 (NH_2_), 3102 (arom. CH), 2209 (CN), 1660 (amide CO) cm^−1^. ^1^H-NMR (DMSO-*d*_6_) *δ* 5.32 (s, 2H, CH_2_), 7.22 (d, 2H, *J* = 6.8 Hz, Ar-H), 7.28 (dd, *J* = 3.4, 3.4 Hz, thiophene-H), 7.31 (t, 1H, *J* = 7.65 Hz, Ar-H), 7.37 (t, 2H, *J* = 7.65 Hz, Ar-H), 7.56 (dd, 1H, *J* = 1.7, 0.85 Hz, thiophene-H), 7.94 (dd, 1H, *J* = 1.7, 1.7 Hz, thiophene-H), 8.45 (br s, 2H, NH_2_). ^13^C-NMR (DMSO-*d*_6_) *δ* 44.85 (CH_2_), 75.63 (C-5), 87.52 (C-3), 115.94 (CN), 116.57 (CN), 126.48 (2Ar-C), 127.44 (1Ar-C), 127.75 (thiophene-C), 128.59 (2Ar-C), 130.51 (thiophene-C), 131.0 (thiophene-C), 133.32 (thiophene-C), 134.37 (1Ar-C), 153.0 (C-6), 156.68 (C-2), 159.44 (C-4). MS: *m*/*z* (%) = 333 (M^+^ + 1, 7), 332 (M^+^, 31), 92 (8), 91 (100), 65 (17); Anal. Calcd. for C_18_H_12_N_4_OS (332.38): C, 65.05; H, 3.64; N, 16.86; S, 9.65. Found: C, 65.15; H, 3.70; N, 16.98; S, 9.4.

*6-Amino-1-butyl-2-oxo-4-(pyridin-3-yl)-1,2-dihydropyridine-3,5-dicarbonitrile* (**4l**). Colorless powder. M.p. 236.6–238.5 °C. IR (KBr) 3509, 3382, 3336 (NH_2_), 3068 (arom. CH), 2958, 2866 (aliph. CH), 2214, 2193 (CN), 1640 (amide CO) cm^−1^. ^1^H-NMR (DMSO-*d*_6_) *δ* 0.92 (t, 3H, *J* = 7.65, CH_3_), 1.32–1.37 (m, 2H, CH_2_), 1.50–1.53 (m, 2H, CH_2_), 4.01 (t, 2H, *J* = 7.65, CH_2_), 7.60 (ddd, 1H, *J* = 6, 6, 0.85 Hz, pyridine-H), 7.97–7.98 (m, 1H, pyridine-H), 8.50 (br s, 2H, NH_2_), 8.70 (dd, *J* = 3.40, 0.85 Hz, pyridine-H), 8.75 (dd, *J* = 6, 0.85 Hz, pyridine-H). MS: *m*/*z* (%) = 294 (M^+^ + 1, 8), 293 (M^+^, 28), 277 (31), 276 (100), 251 (14), 238 (11), 237 (46), 221 (5), 210 (5), 209 (21), 182 (5), 155 (5), 79 (5), 78 (5), 57 (12), 56 (11), 55 (18); Anal. Calcd. for C_16_H_15_N_5_O (293.33): C, 65.52; H, 5.15; N, 23.88. Found: C, 65.44; H, 5.20; N, 24.05.

*6-Amino-1-benzyl-2-oxo-4-(pyridin-3-yl)-1,2-dihydropyridine-3,5-dicarbonitrile* (**4m**). Colorless powder. M.p. 164.4–166.5 °C. IR (KBr) 3527, 3380, 3267, 3114 (NH_2_), 2219 (CN), 1635 (amide CO) cm^−1^. ^1^H-NMR (DMSO-*d*_6_) *δ* 5.34 (s, 2H, CH_2_), 7.24 (d, 2H, *J* = 6.8 Hz, Ar-H), 7.31 (t, 1H, *J* = 6.8 Hz, Ar-H), 7.38 (t, 2H, *J* = 7.65 Hz, Ar-H), 7.62 (dd, 1H, *J* = 7.65, 5.1 Hz, pyridine-H), 8.03 (d, 1H, *J* = 8.5 Hz, pyridine-H), 8.54 (br s, 2H, NH_2_), 8.76 (m, 2H, pyridine-H). ^13^C-NMR (DMSO-*d*_6_) *δ* 44.81 (CH_2_), 75.92 (C-5), 87.86 (C-3), 115.65 (CN), 116.34 (CN), 123.62 (1Ar-C), 126.56 (1Ar-C), 127.50 (1Ar-C), 128.59 (2Ar-C), 130.85 (1Ar-C), 134.32 (pyridine-C), 136.09 (pyridine-C), 148.14 (pyridine-C), 151.28 (pyridine-C), 156.58 (C-6),157.68 (C-2), 159.32 (C-4). MS: *m*/*z* (%) = 328 (M^+^ + 1, 6), 327 (M^+^, 25), 92 (8), 91 (100), 65 (15); Anal. Calcd. for C_19_H_13_N_5_O (327.35): C, 69.71; H, 4.00; N, 21.39. Found: C, 69.82; H, 3.94; N, 21.35.

*6-Amino-1-hexyl-2-oxo-4-(pyridin-3-yl)-1,2-dihydropyridine-3,5-dicarbonitrile* (**4n**). Colorless powder. M.p. 222.9–224.3 °C. IR (KBr) 3364, 3338, 3214 (NH_2_), 2957, 2938, 2859 (aliph. CH), 2220, 2207 (CN), 1648 (amide CO) cm^−1^. ^1^H-NMR (DMSO-*d*_6_) *δ* 0.88 (t, 3H, *J* = 6.8 Hz, CH_3_), 1.32 (m, 6H, 3CH_2_), 1.52 (m, 2H, CH_2_), 3.99 (t, 2H, *J* = 7.65 Hz, N-CH_2_),7.60 (dd, 1H, *J* = 7.65, 5.1 Hz, pyridine-H), 7.98 (m, 1H, pyridine-H), 8.50 (br s, 2H, NH_2_), 8.70 (d, *J* = 1.7 Hz, pyridine-H), 8.75 (dd, *J* = 5.1, 1.7 Hz, pyridine-H). ^13^C-NMR (DMSO-*d*_6_) *δ* 13.97 (CH_3_), 22.03 (CH_2_), 25.58 (CH_2_), 26.16 (CH_2_), 30.99 (CH_2_), 42.18 (N-CH_2_), 75.62 (C-5), 87.75 (C-3), 115.75 (CN), 116.41 (CN), 123.62 (pyridine-C), 130.85 (pyridine-C), 136.03 (pyridine-C), 148.09 (pyridine-C), 151.21 (pyridine-C), 156.20 (C-6), 157.13 (C-2), 159.17 (C-4). MS: *m*/*z* (%) = 322 (M^+^ + 1, 8), 321 (M^+^, 22), 306 (5), 305 (32), 304 (99), 264 (9), 262 (7), 251 (25), 238 (31), 237 (100), 236 (7), 223 (8), 222 (8), 221 (9), 210 (9), 209 (34), 195 (7), 194 (6), 182 (6), 181 (6), 167 (6), 166 (6), 155 (6), 78 (6), 69 (12), 67 (5), 57 (5), 56 (23), 55 (54); Anal. Calcd. for C_18_H_19_N_5_O (321.38): C, 67.27; H, 5.96; N, 21.79. Found: C, 67.36; H, 5.91; N, 21.93.

*6-Amino-1-butyl-2-oxo-4-(pyridin-4-yl)-1,2-dihydropyridine-3,5-dicarbonitrile* (**4o**). Brownish powder. M.p. 313.4–315 °C. IR (KBr) 3358, 3282 (NH_2_), 3090 (arom. CH), 2973, 2958, 2939, 2864 (aliph. CH), 2230, 2209 (CN), 1663 (amide CO) cm^−1^. ^1^H-NMR (DMSO-*d*_6_) *δ* 0.91 (t, 3H, *J* = 7.65 Hz, CH_3_), 1.34 (m, 2H, CH_2_), 1.51 (m, 2H, CH_2_), 4.0 (t, 2H, *J* = 7.65 Hz, N-CH_2_), 7.52 (dd, 2H, *J* = 6, 1.7 Hz, pyridine-H), 8.53 (br s, 2H, NH_2_), 8.79 (dd, 2H, *J* = 6, 1.7 Hz, pyridine-H). ^13^C-NMR (DMSO-*d*_6_) *δ* 13.73 (CH_3_), 19.29 (CH_2_), 28.30 (CH_2_), 41.96 (N-CH_2_), 74.92 (C-5), 87.06 (C-3), 115.43 (CN), 116.08 (CN), 122.50 (2 pyridine-C), 142.43 (pyridine-C), 150.15 (pyridine-C), 150.18 (pyridine-C), 156.23 (C-6), 157.77 (C-2), 159.10 (C-4). MS: *m*/*z* (%) = 294 (M^+^ + 1, 9), 293 (M^+^, 21), 277 (26), 276 (84), 264 (5), 251 (26), 238 (21), 237 (100), 236 (7), 223 (7), 221 (6), 210 (21), 209 (18), 182 (6), 181 (5), 155 (5), 57 (9), 56 (14), 55 (16); Anal. Calcd. for C_16_H_15_N_5_O (293.33): C, 65.52; H, 5.15; N, 23.88. Found: C, 65.69; H, 5.08; N, 23.99.

*6-Amino-1-benzyl-2-oxo-4-(pyridin-4-yl)-1,2-dihydropyridine-3,5-dicarbonitrile* (**4p**). Brownish powder. M.p. 276.3–278.4 °C. IR (KBr) 3357, 3269 (NH_2_), 3088 (arom. CH), 2234, 2208 (CN), 1653 (amide CO) cm^−1^. ^1^H-NMR (DMSO-*d*_6_) *δ* 5.32 (s, 2H, CH_2_), 7.25 (d, 2H, *J* = 7.65 Hz, Ar-H), 7.31 (t, 1H, *J* = 7.65 Hz, Ar-H), 7.38 (t, 2H, *J* = 7.65 Hz, Ar-H), 7.57 (d, 2H, *J* = 5.1 Hz, pyridine-H), 8.79 (d, *J* = Hz, 2 pyridine-H). ^13^C-NMR (DMSO-*d*_6_) *δ* 44.71 (CH_2_), 75.56 (C-5), 86.38 (C-3), 115.65 (CN), 11629 (CN), 122.57 (2 pyridine-C), 126.64 (2 Ar-C), 127.41 (1 Ar-C), 128.54 (2 Ar-C), 134.64 (1 Ar-C), 142.61 (pyridine-C), 150.16 (2 pyridine-C), 156.71 (C-6), 158.0 (C-2), 159.50 (C-4). MS: *m*/*z* (%) = 328 (M^+^ + 1, 6), 327 (M^+^, 25), 92 (8), 91 (100), 65 (15); Anal. Calcd. for C_19_H_13_N_5_O (327.35): C, 69.71; H, 4.00; N, 21.39. Found: C, 69.59; H, 4.07; N, 21.21.

*6-Amino-1-hexyl-2-oxo-4-(pyridin-4-yl)-1,2-dihydropyridine-3,5-dicarbonitrile* (**4q**). Brownish powder. M.p. 327.5–328.7 °C. IR (KBr) 3352, 3280 (NH_2_), 2956, 2930, 2854 (aliph. CH), 2227, 2208 (CN), 1656 (amide CO) cm^−1^. ^1^H-NMR (DMSO-*d*_6_) *δ* 0.88 (t, 3H, *J* = 7.65 Hz, CH_3_), 1.30 (m, 6H, 3 CH_2_), 1.52 (m, 2H, CH_2_), 3.99 (t, 2H, *J* = 7.65 Hz, N-CH_2_), 7.52 (dd, 2H, *J* = 6, 1.7 Hz, pyridine-H), 8.53 (br s, 2H, NH_2_), 8.78 (dd, 2H, *J* = 6, 1.7 Hz, pyridine-H). ^13^C-NMR (DMSO-*d*_6_) *δ* 13.96 (CH_3_), 22.02 (CH_2_), 25.56 (CH_2_), 26.12 (CH_2_), 30.97 (CH_2_), 42.2 (N-CH_2_), 74.91 (C-5), 87.07 (C-3), 115.43 (CN), 116.08 (CN), 122.50 (2 pyridine-C), 142.43 (pyridine-C), 150.16 (1 pyridine-C), 150.18 (1 pyridine-C), 156.22 (C-6), 157.77 (C-2), 159.09 (C-4). MS: *m*/*z* (%) = 322 (M^+^ + 1, 6), 321 (M^+^, 16), 305 (23), 304 (75), 264 (7), 262 (6), 251 (25), 238 (27), 237 (100), 210 (12), 209 (12), 69 (10), 56 (20), 55 (41); Anal. Calcd. for C_18_H_19_N_5_O (321.38): C, 67.27; H, 5.96; N, 21.79. Found: C, 67.21; H, 5.92; N, 21.69.

## 4. Conclusions

In summary, we have developed a novel, facile, efficient, rapid, and environmentally friendly approach for the one-pot multicomponent synthesis of new diversely substituted 6-amino-2-oxo-pyridine-3,5-dicarbonitrile derivatives from simple and readily available diverse aldehydes, various *N*-alkyl-2-cyanoacetamides and malononitrile in the presence of K_2_CO_3_ under heating or under microwave activation. The ease of work-up, rapid access, general applicability, greenness of procedure and high isolated yields of products make this new strategy a very useful addition to modern synthetic methods and attractive for academic research and potential applications. Further exploration of the reaction scope and synthetic applications of this methodology are currently under studying in our laboratory.

## Figures and Tables

**Table 1:** Formation of compounds **4a**–**q** under thermal and microwave irradiation.

No.	Heat Time		µω Time	
	(min)	Yield (%)	(min)	Yield (%)
**4a**	60	70	10	91
**4b**	90	77	10	94
**4c**	90	71	10	87
**4d**	90	73	10	91
**4e**	90	76	10	92
**4f**	90	70	10	88
**4g**	90	65	10	81
**4h**	180	69	12	90
**4i**	90	67	10	83
**4j**	180	71	12	87
**4k**	120	73	11	92
**4l**	180	69	15	85
**4m**	180	72	13	93
**4n**	240	70	15	83
**4o**	120	72	11	88
**4p**	120	75	11	92
**4q**	120	71	11	85
